# Advances of aptamers in esophageal cancer diagnosis, treatment and drug delivery

**DOI:** 10.3762/bjnano.16.121

**Published:** 2025-10-06

**Authors:** Yang Fei, Hui Xu, Chunwei Zhang, Jingjing Wang, Yong Jin

**Affiliations:** 1 School of Pharmaceutical Sciences, Anhui Medical University, Hefei, Anhui, China

**Keywords:** aptamers, detection, drug delivery, esophageal cancer, esophageal squamous cell carcinoma, therapy

## Abstract

Esophageal cancer (EC) is a common malignant tumor of the digestive tract with poor prognosis and high mortality. The early diagnosis of EC mainly depends on endoscopic diagnosis, which not only needs to bear certain economic pressure, but also needs patients to recognize the high risk factors of EC. Most EC patients are diagnosed at intermediate or late stages, often due to a lack of awareness regarding early symptoms and lifestyle-related risk factors. However, the discovery of aptamers and the development of nanocarriers bring great benefits to the diagnosis, treatment, and targeted drug delivery of EC. Aptamers or peptide aptamers as biosensors or therapeutic agents for the diagnosis or treatment of EC, aptamer–drug conjugates and aptamer-functionalized drug nanocarriers for targeted drug delivery in esophageal cancer are reviewed in this paper. In addition, we expect investigators to pay special attention to improving aptamer permeability and stability to accelerate aptamer clinical transformation. In conclusion, leveraging the high target specificity of well-selected aptamers may bring new breakthroughs in the diagnosis, treatment and drug delivery of EC.

## Review

### 1 Introduction

**Figure 1:**
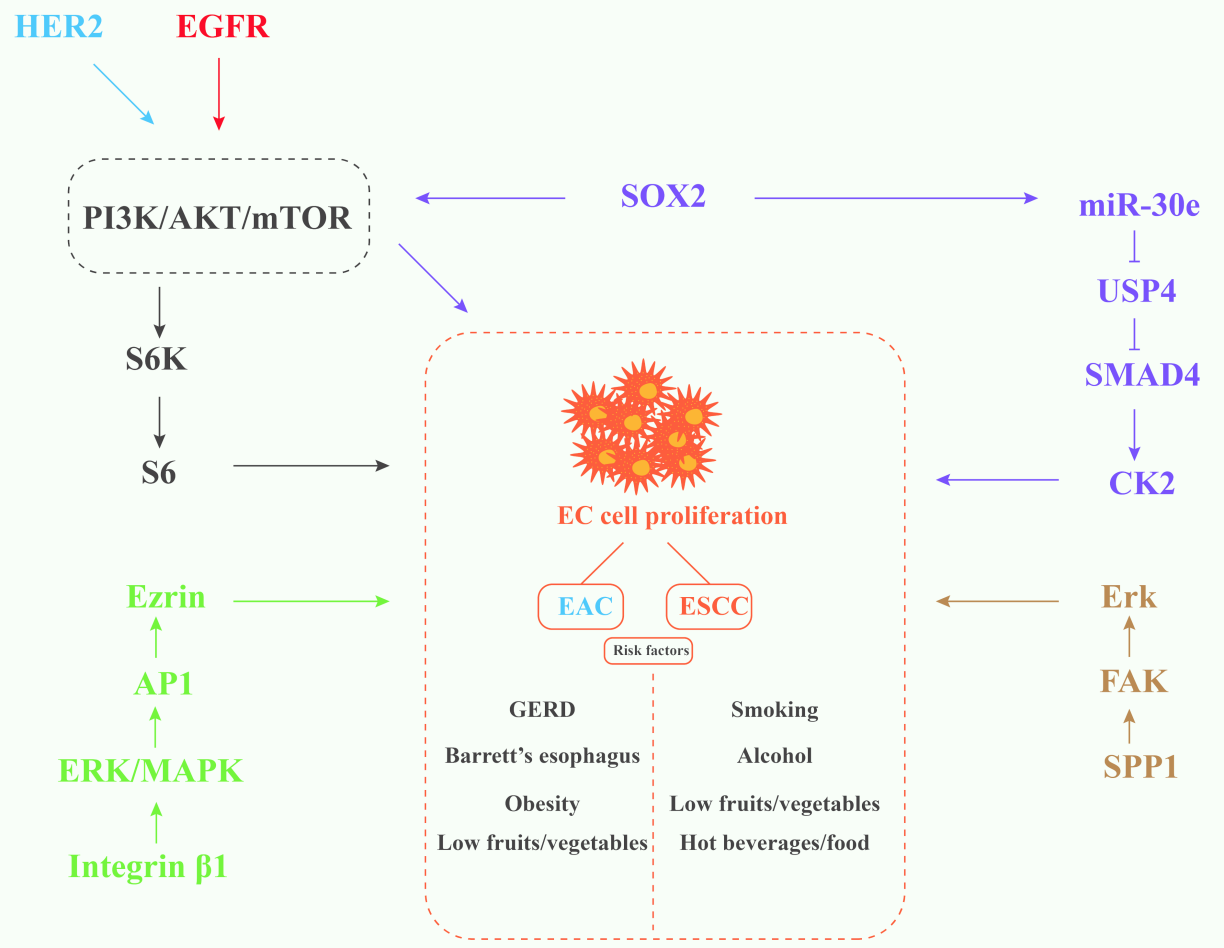
Schematic diagram of some signaling pathways and risk factors that promote esophageal cancer progression. Overexpression of epidermal growth factor receptor (EGFR), SRY-Box Transcription Factor 2 (SOX2), integrin beta 1 (Integrin β1), and secreted phosphoprotein 1 (SPP1) proteins can be observed in ESCC, while EAC mainly shows overexpression of human epidermal growth factor receptor 2 (HER2) protein.

Esophageal cancer (EC) is listed as the seventh leading cause of cancer death, and the pathological types mainly include esophageal squamous cell carcinoma (ESCC) and esophageal adenocarcinoma (EAC). The former is the most common type of primary esophageal cancer, which occurs mostly in elderly men with smoking and drinking habits, while obesity and long-term gastroesophageal reflux disease (GERD) [1] are key factors leading to the occurrence of the latter. In EC, the activation or regulation of pathways such as integrin β1/MAPK/ERK/AP-1 pathway [2], SPP1/FAK/Erk pathway [3], EGFR/PI3K/AKT/mTOR/S6 pathway [4], HER2/PI3K/AKT/mTOR/S6 pathway [5], and SOX2/miR-30e/USP4/SMAD4/CK2 pathway [6] are important mechanisms promoting the malignant phenotype of cancer cells. Some of the pathogenesis and risk factors of ESCC and EAC are shown in Figure 1. The initial clinical manifestations of both conditions are often nonspecific and subtle, frequently escaping clinical detection. Definitive diagnosis typically requires upper gastrointestinal endoscopy to identify potential early neoplastic changes [7–8]. Barrett’s esophagus is generally considered to be a precancerous lesion of EAC [9–10], while esophageal intraepithelial neoplasia is a precancerous stage of ESCC [11–12]. However, the cost burden of general endoscopic screening is extremely high, and some predictive models [13–15] used in stratified screening still need to be optimized and verified in terms of cost-effectiveness. Recent advancements by Chinese researchers have led to the development of a noninvasive esophageal cancer screening method utilizing cfDNA methylation analysis [16]. While this innovation addresses the critical need for population-scale screening in China, its widespread implementation may be constrained by substantial economic costs. Furthermore, immunotherapy has emerged as a prominent therapeutic modality in esophageal cancer management, demonstrating considerable clinical promise in recent years. Several clinical trials [17–18] have emphasized the advantages of immune checkpoint inhibitors over conventional chemotherapy. Meanwhile, targeted agents targeting epidermal growth factor receptor (EGFR), human epidermal growth factor receptor 2 (HER2), and vascular endothelial growth factor receptor can also overcome the limitations of the high incidence of adverse events in traditional radiotherapy and chemotherapy models [19]. However, due to primary resistance and acquired resistance, most EC patients cannot benefit from immunotherapy [20–21]. Among the many targeted drugs, only trastuzumab, papolizumab, and remolizumab have been approved by the FDA for the treatment of EC [22]. In addition, small-molecule chemotherapeutic agents are also an important part of esophageal cancer treatment [23] and are considered to have some immune activation effects [24]. However, the unavoidable drug resistance problems of small-molecule drugs, their fast clearance rate, and a series of side effects caused by nonspecific use are serious obstacles to their safe application. Hence, ongoing research explores strategies to optimize the solubility and targeting ability of anti-EC drugs, and aptamers [25] represent a distinct class of molecular tools.

Aptamers are small nucleotide or peptide sequences screened by “systematic evolution of ligands by exponential enrichment” (SELEX) technology, and their folded three-dimensional structure realizes high affinity and specific binding with targets; also, they have the advantages of relatively small molecular size, cost-effective production, broad target spectrum, and high adaptability for structural modification. [26]. Aptamer-based biosensors [27] fully utilize these characteristics by combining various signal amplification techniques and nanomaterials (such as metal nanoparticles, quantum dots, silica nanoparticles, and carbon nanotubes), enabling highly sensitive detection of biomarkers. These sensors feature minimal invasiveness and low detection limits, demonstrating unique advantages in the early detection of cancer biomarker proteins. Moreover, an advanced drug delivery platform [28] was engineered by functionalizing nanocarriers with the AS1411 aptamer, enabling the targeted co-delivery of a P-glycoprotein (P-gp) inhibitor, specifically, small interfering RNA (siRNA) against the MDR1 gene, along with the chemotherapeutic agent doxorubicin (DOX). This system capitalizes on the high-affinity binding of the AS1411 aptamer to nucleolin, a protein overexpressed on the surface of cancer cells, thereby significantly enhancing tumor-specific drug accumulation. Concurrently, siRNA-mediated silencing of MDR1 effectively suppresses P-gp-mediated drug efflux, overcoming multidrug resistance (MDR) in tumor cells. By integrating active targeting, gene silencing, and chemosensitization, this synergistic strategy presents a promising approach to circumvent chemotherapy resistance in cancer treatment.

In general, DNA aptamers have higher thermal stability, RNA aptamers are richer in secondary structure, and peptide aptamers are smaller in size and easier to enter cells [29]. Furthermore, peptide aptamers exhibit target specificity predominantly limited to protein molecules, representing a relatively constrained target spectrum. Nucleic acid aptamers, which can bind to proteins, genes, small molecules, cells and other targets, are commonly used in laboratory and clinical practice and further play a role in diagnosis and therapy by identifying and regulating the expression level of targets. In addition, SELEX has also been further developed into cell-SELEX [30], subtractive EMSA-SELEX [31], and CE-SELEX [32] along with technological progress, which is helpful to improve screening efficiency.

However, only a small fraction of nucleotides of the full-length aptamers obtained by SELEX are critical for interacting with the target protein. Given the propensity of non-essential nucleotide regions to induce steric constraints and nonspecific binding, systematic bioinformatic analysis through tools like Mfold is essential for optimizing aptamer truncation while preserving target affinity [33]. Also, unmodified aptamers are susceptible to nuclease degradation and renal filtration. It is often necessary to improve the retention and efficacy of aptamers in vivo by modifying nanocarriers, coupling drugs, covalent binding with siRNA, and chemical modification [34–35] to promote their role in cancer drug delivery. Conventional drug delivery systems, including metal nanoparticles, nanohydrogels, liposomes, and polymeric micelles [36–38], have gained widespread adoption due to their optimal biocompatibility, structural stability, and superior drug-loading capacity. However, nanocarriers are prone to complement system recognition [39] and clearance due to their microbial-scale dimensions and surface-exposed nucleophilic groups, which inadvertently trigger immune activation and contribute to off-target effects. This fundamental limitation explains the persistent observation of diverse adverse effects in both preclinical animal studies and clinical applications of carrier-modified therapeutics. If aptamer modification is carried out on the surface of the carrier [40], it will help to improve the penetration and accumulation of drugs in the lesion, providing more possibilities for the treatment strategy of EC. In addition, aptamer-based biosensors offer flexibility in design, combined with nanotechnology to achieve outstanding sensitivity [41]. Detection of EC-related biomarkers through this dual action [42–43] provides a powerful tool for early diagnosis of EC.

Aptamers have brought novel contributions and developments in diagnosis, treatment, and drug delivery of EC in recent years. The aim of this paper is to elucidate the role of aptamers as biosensors, targeted therapeutics and targeted delivery agents to provide more accurate, efficient and safer diagnosis and treatment options for EC patients. Initially, this review describes the high selectivity and binding ability of aptamers to specific EC markers, showing great potential for early detection of EC and monitoring of therapeutic efficacy. Recent breakthroughs in aptamer-based targeted therapeutics have marked significant advancements, revitalizing prospects for novel therapeutic aptamer developments. In addition, aptamer-based drug delivery systems can not only accurately identify lesions, but also improve drug loading and release efficiency with nanomaterials as carriers, significantly optimizing the treatment mode of EC.

### 2 Searching core literature

**Figure 2:**
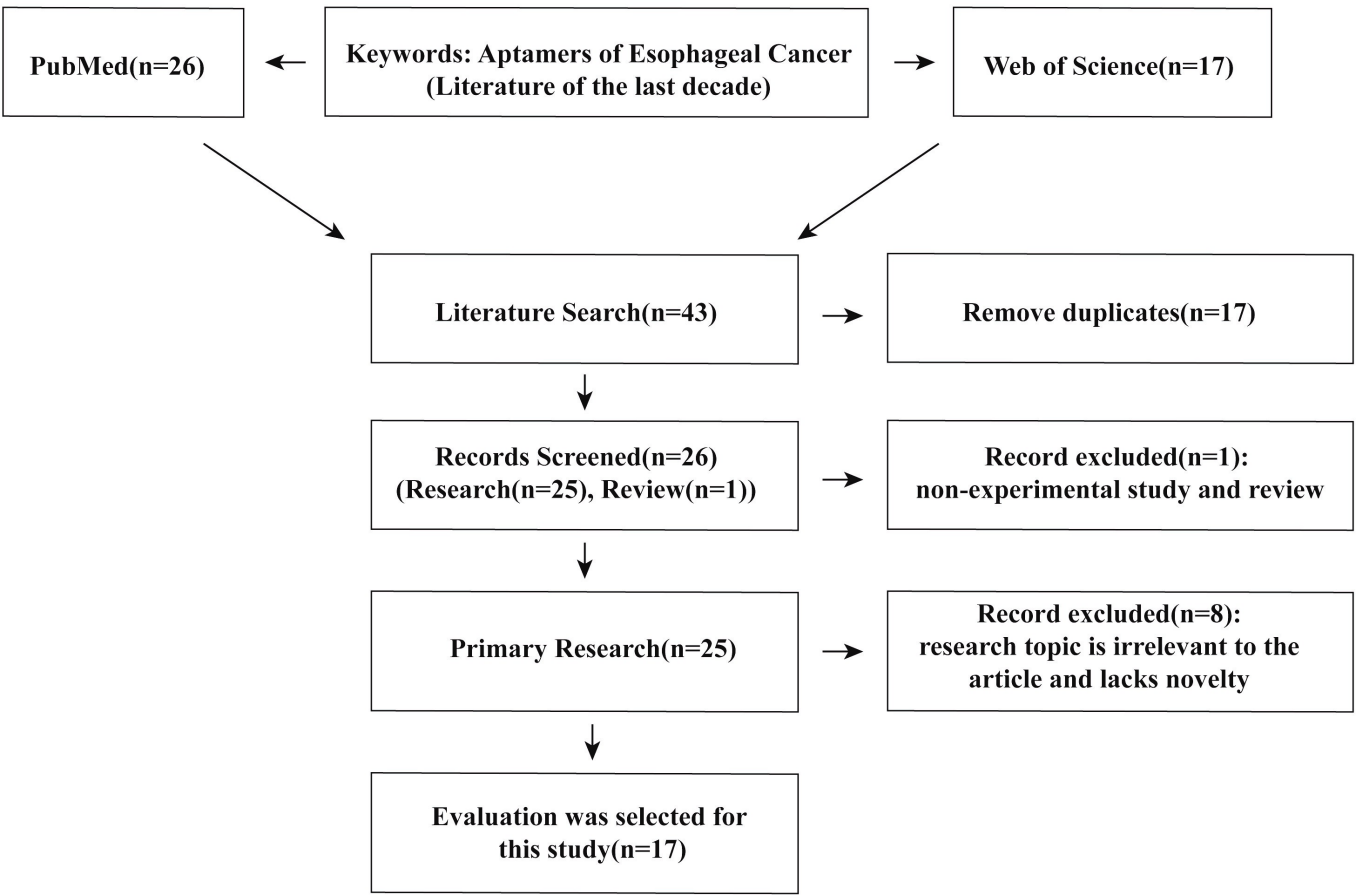
Flowchart of literature selection.

In order to better summarize the application of aptamers in the diagnosis, treatment, and drug delivery of EC, we searched for keywords in the PubMed and Web of Science databases: “Aptamers of Esophageal Cancer”. The inclusion criteria are: (1) literature from the last decade (2015–2025); (2) the studies have said that the introduction of aptamers provides a promising strategy for the diagnosis, treatment, and drug delivery of esophageal cancer; and (3) selection, synthesis, and technical details of aptamers and their applications are described in detail in the article. The exclusion criteria are: (1) review, letter, news; (2) comment of a conference or seminar; (3) case report; or (4) irrelevant research topic of the article. Eventually, 17 original research articles on aptamers for EC diagnosis, treatment, and drug delivery were selected. The selecting process of the literature is shown in Figure 2.

### 3 Aptamers as biosensors: diagnosis of esophageal cancer

**Figure 3:**
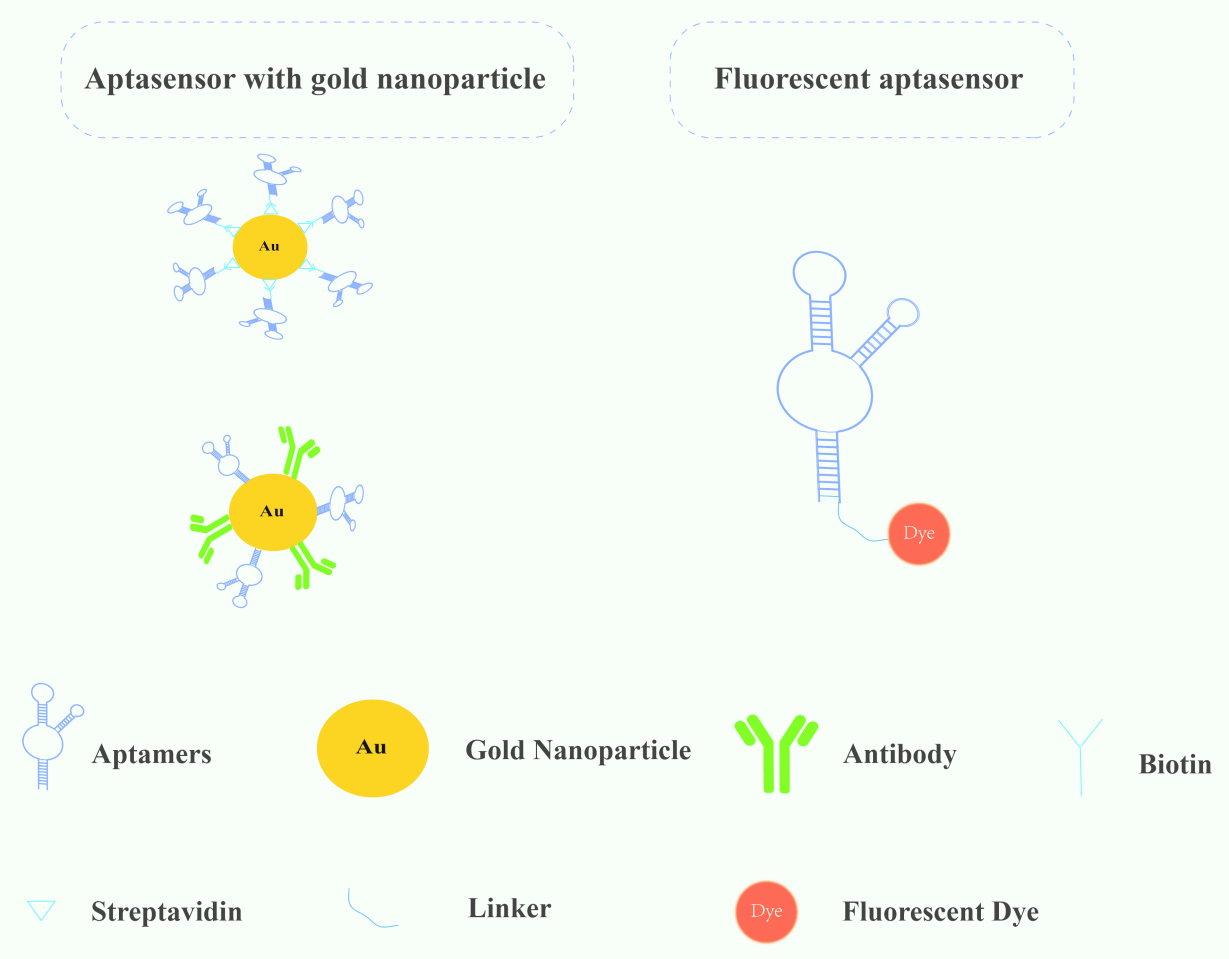
Schematic diagram of gold nanoparticle aptasensor and fluorescent aptasensor for esophageal cancer diagnosis.

Biomarkers are measurable biological indicators that exhibit pathological alterations during disease progression, primarily encompassing proteins, nucleic acids, metabolites, and cellular components [44]. To be clinically relevant, these markers must demonstrate both high sensitivity and specificity, enabling robust diagnostic and prognostic capabilities. Most biosensors for cancer detection use a single biometric element to directly analyze biomarkers [45–46], while dual-system biosensors combine two antibodies and/or aptamers [47] into a better composite material to improve the specificity and sensitivity of the sensor. Figure 3 is a schematic diagram of a gold nanoparticle aptasensor and a fluorescent aptasensor for the diagnosis of EC. Scholars have also found significant differences in conclusions among studies, taking tumor heterogeneity into account and raising expectations that studies based on multiple markers [48] acting on different pathways may have more specific diagnostic outcomes. The establishment of a multiprotein model [49] confirms this conjecture. There are already gold nanoparticle aptamer biosensors and fluorescent aptamer sensors that show great potential in esophageal cancer diagnosis (Table 1).

**Table 1:** Aptamers for esophageal cancer detection.

Target	Aptamer	Sequence(5′–3′)	Target cell line	Preclinical model	Refs.

EGFR	EGFR-Aptamer	UGC CGC UAU AAU GCA CGG AUU UAA UCG CCG UAG AAA AGC AUG UCA AAG CCG	Eca109	–	[53–54]
EGFR	EGFR-Aptamer	CCG CTT TAT TGT TAA TTA AGT TTT ATA TTT CGCA CAA CACACA ACA ATC AAT ATC	Eca109	–	[55]
HER2	HER2-Aptamer	GCA GCG GTG TGG GGG CAG CGG TGT GGG GGC AGC GGT GTG GGG TTT TT	Eca109	–	[55]
unknown	S3-2-3	ATG GCC AGG GGG GAG TGGG	KYSE 150	–	[56]
EpCAM	SYL3C	CAC TAC AGA GGT TGC GTC TGT CCC ACG TTG TCA TGG GGG GTT GGC CTG	– (patient samples)	– (patient samples)	[57]

#### 3.1 Gold nanoparticle–aptamer sensors

Scholars reviewed eleven immunohistochemical prognostic markers including EGFR in ESCC-related literature and evaluated the prognostic value of individual markers [50]. Overexpression of EGFR protein is common in ESCC patients and is significantly associated with tumor aggressiveness, lymph node metastasis, and poor prognosis [51]. Therefore, the detection of EGFR biomarkers by developing simple and efficient methods may become an important strategy for early diagnosis of ESCC.

Ilkhani et al. [52] designed an electrochemical sandwich immunosensor. The anti-EGFR aptamer was immobilized on magnetic beads, and the anti-EGFR antibody was combined with gold nanoparticles to form a complex. The target EGFR in test samples was specifically captured by the aptamer, subsequently forming a sandwich detection complex. While the preparation and detection workflow demonstrates notable simplicity, its clinical performance was rigorously validated using samples from breast cancer patients. Li et al. [53] used positively charged gold nanoparticles modified with anti-EGFR aptamer and anti-EGFR antibody (Aptamer-AuNPs-Ab) as an immunosensor to sensitively and specifically detect EGFR concentrations in Eca109 cell lysates and human serum samples. The capture probe is an EGFR aptamer with thiol modification, and the signal probe is an anti-EGFR antibody. This multifunctional cellular probe specifically recognizes and binds to EGFR on the cell surface, inducing the formation of probe aggregates that significantly enhance resonance Rayleigh scattering (RRS) signals. This strategy can be extended to the detection of other biomarkers simply by changing the ligand. For low-abundance markers, the signal amplification effect of gold nanoparticles [54] may show the huge advantages of Aptamer-AuNPs-Ab. Later, the same research team [55] reported a sandwich scattering system based on dual aptamers targeting EGFR and HER2. This design not only capitalizes on the overexpression characteristics of EGFR/HER2 in EC (as discussed in the Introduction), but also enhances detection sensitivity through the synergistic effect of dual aptamers, directly addressing the clinical targeting needs highlighted in the aforementioned signaling pathways. RRS analysis showed that the hybrid probe (probe I–cell–probe II) was more sensitive to marker determination than a single probe, while improving detection of cells expressing both markers. The authors are still in the process of optimizing the system parameters to make the method meet the needs of clinical sample testing. However, there may be some endogenous biotin-binding proteins or exogenous uptake of biotin in clinical samples from esophageal cancer patients that may bind nonspecifically to this system, resulting in false positive diagnoses. Furthermore, the diagnostic accuracy achieved through resonance RRS analysis may be inferior to that of enzyme-linked immunosorbent assay methodologies.

#### 3.2 Fluorescent aptasensors

Chen et al. [56] screened and optimized the sequence by Cell-SELEX to obtain nucleic acid aptamer S3-2-3 with binding specificity to ESCC cells. After labeled with Cy5 dye, it can yield highly specific fluorescence imaging for ESCC tissues, providing accurate display tools for clinical diagnosis. The remarkably short 18-nucleotide length of aptamer S3-2-3 enables its development as a cost-effective, yet highly specific, molecular probe. Mass spectrometry analysis confirmed its target as a membrane-associated protein. Another study [57] showed that EpCAM was overexpressed in esophageal cancer samples, and the expression level of EpCAM was detected using antibodies or aptamers. After Cy3 staining, the results showed that the two staining results were similar. The imaging method of the aptamer is simpler, indicating that the aptamer SYL3C can be used as a molecular diagnostic tool instead of antibody.

Although there are many potential biomarkers for ESCC, and noninvasive methods are the trend in large-scale screening, multiple challenges such as marker selection, cost, and individual differences [58] have hindered their clinical translation. The emergence of spatial transcriptomics technology [59] offers a promising solution to overcome this current methodological limitation. By exploring deeper spatial patterns of gene expression [60], combined with single-cell RNA sequencing [61], it increases our understanding of the full range of ESCC mechanisms. Genomic studies of ESCC and EAC [62] revealed significant differences in marker expression, possibly indicating different treatment strategies.

### 4 Aptamers as targeted esophageal cancer therapeutics

**Figure 4:**
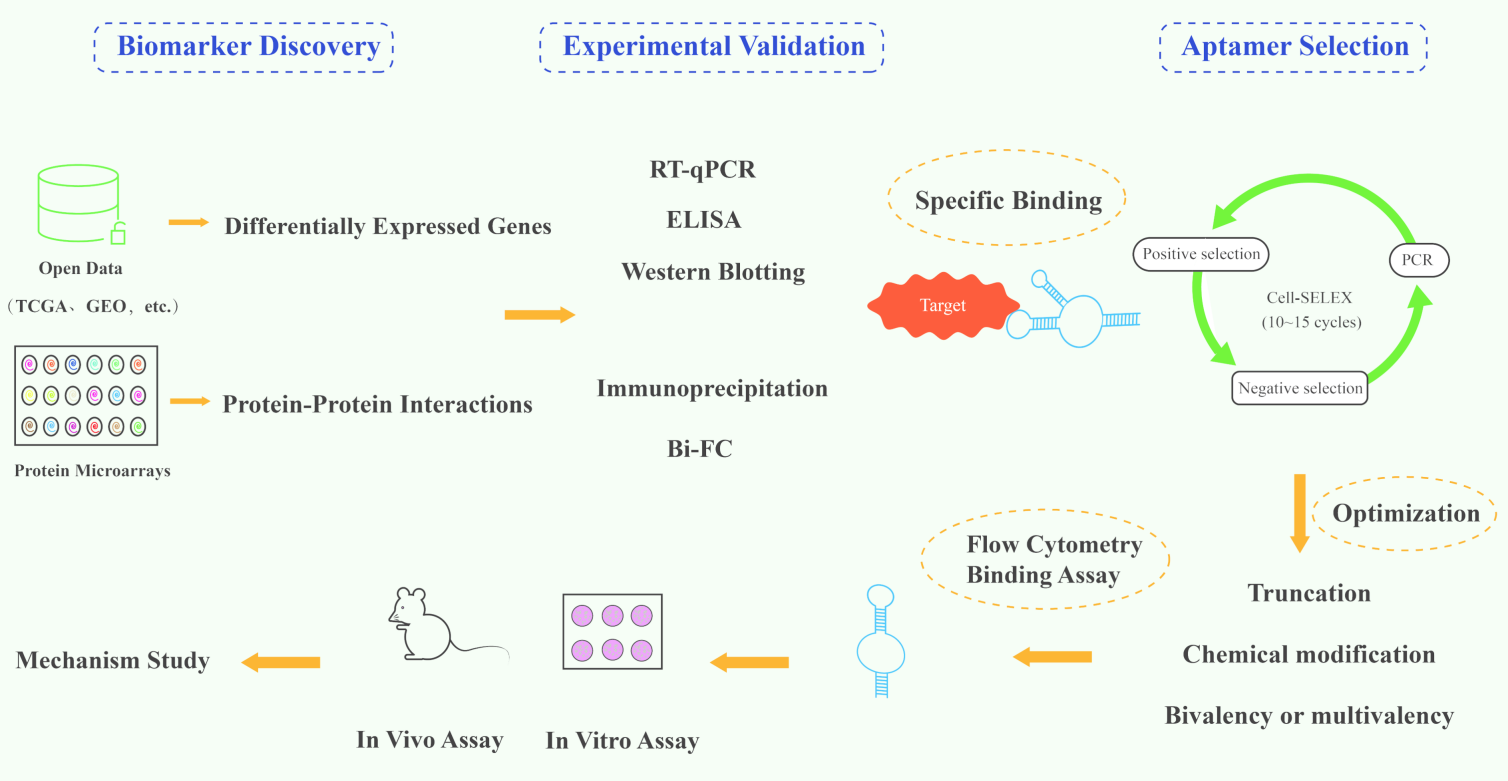
Schematic diagram of the general research and development process of aptamers for the treatment of esophageal cancer.

As of 2024, aptamer-based therapeutics remain limited in clinical use, typically represented by the DNA aptamer Pegaptanib and the RNA aptamer Avacincaptad pegol. In December 2004, Pegaptanib [63], the first aptamer as clinical therapeutic agent, was approved by FDA for the treatment of age-related macular degeneration, which pioneered the use of aptamer drugs in clinical treatment. In August 2023, another aptamer therapeutic agent, Avacincaptad pegol [64], was approved by the FDA for the treatment of geographic atrophy. Pegaptanib and Avacincaptad pegol can be administered topically by intravitreal injection, easily achieving ideal bioavailability. However, the clinical utility of aptamer-based therapeutics remains limited. Developing new aptamers for esophageal cancer therapy presents multiple challenges, such as lack of clear targets, off-target effects, poor stability, and difficulty in penetrating cell membranes. For therapeutic aptamer development, researchers typically: (1) identify dysregulated proteins or molecular targets in patient-derived cancer specimens; (2) select target-specific aptamers through systematic evolution of ligands by SELEX; (3) validate binding affinity and functional modulation in vitro and in vivo models; and (4) elucidate the underlying molecular mechanisms (Figure 4). At present, significant progress has been made in peptide aptamers targeting SOX2 protein or its binding partner, aptamers targeting *Mycoplasma hyorhinis* and aptamers targeting SPP1 (Table 2).

**Table 2:** Aptamers in the targeted therapy of esophageal cancer.

Target	Aptamer	Sequence (5′–3′)	Target cell line	Preclinical model	Refs.

SOX2	P42	FSTLFFPLFFL	KYSE450	BALB/c nude mice	[75]
CDP/SOX2	P58	YLFAIYSFSSL	KYSE450 and KYSE30	BALB/c nude mice	[80]
p37/TLR4	ZY3A	A*C*C *G*AC CGT GCT GGA CTC ACG TCG TCC ATT TCC TTG AAA AAG GCA CGG GTT CCA TGA ACT CAC TAT *G*A*G *C-invert dT * phosphorothioate modified	Eca109 and KYSE150	BALB/c nude mice	[91]
SPP1	SPP1 aptamer	CGG CCA CAG AAU GAA AAA CCU CAU CGA UGU UGC AUA GUU G	KYSE150	BALB/c nude mice	[100]

#### 4.1 Peptide aptamers targeting SOX2 protein or its binding partners

Peptide aptamers consist of amino acid sequences and can be screened by yeast two-hybrid [65], phage display [66], or molecular docking prediction techniques [67]. The first peptide aptamer was proposed in 1996 to recognize and inhibit cyclin dependent kinase 2 (Cdk2) [68]. Later, peptide aptamers were used directly as drugs to regulate proteins and genes [69–71], showing potential in the field of targeted cancer therapy. Transcription factor SOX2, as a lineage survival oncogene of ESCC [72], can maintain the squamous characteristics of tumor cells and is essential for the proliferation and survival of esophageal cancer cell lines. Moreover, SOX2 and DMRTA1 (DMRT-like family A1) can promote the expression of each other, which may drive the occurrence of ESCC resistance [73]. Notably, transcription factors like SOX2 are typically categorized as “non-druggable targets” due to three fundamental challenges: (1) their structurally flat DNA-binding domains, (2) extensive protein–protein interaction interfaces, and (3) the absence of well-defined binding pockets for conventional small molecules [74]. These characteristics collectively hinder the development of effective small-molecule inhibitors that can specifically bind and functionally inhibit these targets. In contrast, nucleic acid aptamers overcome these limitations through their unique capacity to form intricate three-dimensional structures that specifically recognize and bind to SOX2. This binding effectively disrupts SOX2–DNA interactions, thereby establishing a novel therapeutic strategy for traditionally undruggable targets. Most recently, researchers have developed peptide-based aptamers targeting SOX2 that demonstrate promising therapeutic potential, as evidenced by their successful suppression of downstream oncogenic pathways in tumor models. Liu et al. [75] demonstrated that SOX2 exhibits significantly high expression in ESCC tissues using the clinical sample data of 75 ESCC cases, and screened peptide aptamer p42, which can inhibit the proliferation, migration, and invasion of ESCC tissues. It was confirmed that synthetic peptide 42 similarly inhibits SOX2 in vitro and in vivo, which may overcome drug resistance to some extent. Proteomic analysis revealed significant changes in the levels of several proteins associated with tumor progression, especially SQRDL and MAU2, while SOX2 levels were almost unaffected. Although there are other strategies that can achieve selective degradation of SOX2, SOX2 lacks pockets suitable for small-molecule drug binding, and proteolytically targeted chimeras are required to degrade SOX2-like “non-druggable” targets [76–77]. In addition, some literature points out that directly targeting SOX2 is inappropriate and may cause multiple complications [78].

As our understanding of disease deepens, a series of attractive new drug targets, such as protein–protein interactions [79], begin to emerge. Chen et al. [80] showed that CDP and SOX2 expression levels were highly correlated with late ESCC, verified that CDP and SOX2 interacted in ESCC cell lines (KYSE450 and KYSE30 cells), and confirmed the interaction interface between these two proteins. Peptide aptamer p58, which can specifically block CDP/SOX2 interaction, was screened by BiFc assay. The antitumor activity of p58 aptamer was further analyzed in vitro and in mouse xenograft and zebrafish models, suggesting that peptide 58 could significantly inhibit the proliferation, migration, and invasion of KYSE450 cells, slow down tumor growth and metastasis, and had no obvious toxicity to human normal esophageal epithelial cells. Cluster analysis showed that the mechanism of action of peptide 58 may be closely related to the inhibition of ESCC cell metabolism. However, pharmacokinetic data on peptide 58 remain unclear.

#### 4.2 Aptamers targeting *Mycoplasma hyorhinis*

*Mycoplasma hyorhinis* is a prevalent swine pathogen that primarily colonizes the porcine middle ear and nasal cavity [81]. This organism is clinically significant as it can induce a range of pathological manifestations, including polyarthritis-associated lameness, otitis media, and serositis [82]. Current therapeutic interventions predominantly rely on antimicrobial agents and prophylactic vaccination strategies [83]. *Mycoplasma hyorhinis* infection has also been reported to have the potential to indirectly cause hepatocellular carcinoma [84], gastric cancer [85], prostate cancer [86], and ovarian cancer [87]. Scientists have discovered that p37 protein promotes invasiveness and induces malignant transformation of cells [88], which may be a key target for *Mycoplasma* pathogenicity. However, given that *Mycoplasma*-mediated cellular malignant transformation is characterized by long-latency, multistage chronic infections [89], it is extremely challenging to collect sufficient epidemiological data to establish a direct causal relationship between *Mycoplasma* infection and tumors. Immunohistochemical results of 53 cancer tissue samples showed that *Mycoplasma* infection was 50.9% positive in EC tissue, and there was a high correlation between *Mycoplasma* infection and EC tissue [90]. Zhang et al. [91] showed that *M. hyorhinis* was closely related to migration, invasion, and metastasis of ESCC tissues, and thus DNA aptamer ZY3 against *M. hyorhinis* and the optimized truncation ZY3A with stronger binding ability were developed. The binding target was p37 protein, with *K*_D_ = 4.75 ± 1.16 nM. In vivo and in vitro experiments showed that ZY3A has targeting ability to *Mycoplasma hyorhinis*-infected cells, and its therapeutic effect in vitro is equivalent to that of azithromycin, but its effect in vivo is inferior. It is expected that ZY3A can be applied to clinical practice through optimizing drug administration strategies, such as combination of antibiotics and improvement of the aptamer structure. The mechanism of action of ZY3A may be to inhibit NF-κB pathway and MMP2 levels by blocking the interaction between p37 and TLR4. ZY3A is also considered to have a broad spectrum and can target other *M. hyorhinis*-positive tumor cells; the corresponding molecular mechanisms and receptor proteins involved deserve further study and exploration. In addition, ZY3A is of low molecular weight, easy to excrete by the kidneys, and needs to be further modified as a drug carrier [92].

#### 4.3 Aptamers targeting SPP1

Secreted phosphoprotein 1 (SPP1), also known as osteopontin (OPN), is a secreted phosphorylated glycoprotein [93] that binds to integrins and CD44 mainly through three domains, including RGD [94], and participates in cell adhesion, migration, proliferation, and signal transduction [95]. Five subtypes of OPN are co-upregulated in primary EAC, among which OPNc expression was relatively low, inducing cell separation via an integrin-independent binding mode, probably due to its deletion of the key RGD sequence binding to integrin [96]. Based on an analysis of 171 clinical samples, 120 common differentially expressed genes were identified in esophageal tumor samples. Further genetic evaluation determined that SPP1 expression was a strong independent predictor of OS, and its high expression was significantly associated with poor prognosis [97]. SPP1 is also thought to be a pivotal gene that may play a key role in ESCC progression [98]. In addition, SPP1 overexpression in tumor cells causes the immune system to respond to SPP1 and produce anti-SPP1 autoantibodies [99], which may make anti-SPP1 autoantibodies a novel serum biomarker for detecting ESCC patients. Wang et al. [100] found that SPP1 can recruit macrophages and activate the CD44/PI3K/AKT signaling axis, which in turn promotes their polarization to the M2 type, creating a microenvironment conducive to tumor growth and promoting ESCC progression. RNA aptamers targeting SPP1 upregulate the expression of M1-like markers, reduce the number and function of M2 tumor-associated macrophages, inhibit ESCC progression, and prolong survival. Interestingly, in mice depleted of peritoneal macrophages, knockout of SPP1 no longer reduced tumor volume. This suggests that the presence of macrophages is largely required for SPP1-deleted antitumor effects and provides new ideas for developing combination therapy strategies against SPP1 and macrophages, such as SPP1 small-molecule inhibitors, RNA aptamers for SPP1, siRNA, shRNA [101], and depletion and reprogramming drugs for M2-like macrophages [102–103].

### 5 Aptamers in targeted drug delivery against esophageal cancer

Aptamer–drug conjugates (ApDCs) are capable of precisely delivering drugs to target cells or tissues using aptamers as “missiles” [104]. The high specificity and affinity of aptamers make them ideal targeting vectors for efficient delivery of multiple types of therapeutic agents to specific lesion sites. Therapeutic cargoes for drug modification extend beyond conventional chemotherapeutic agents to encompass emerging gene-modulating therapeutics, including siRNA, microRNA, and other nucleic acid-based pharmaceuticals. It should be noted that designing aptamer–siRNA conjugates requires special attention to overcome nuclease degradation and improve the pharmacokinetic parameters of the system [105]. At present, therapeutic antibodies are far ahead of aptamers in terms of global market share. However, the large size of antibodies (150–180 kDa, 15 nm), limited tissue permeability, expensive, time-consuming and laborious in vivo screening, and high immunogenicity [26] make it imperative to develop safe, effective, and simple ApDCs.

Aptamer-functionalized drug nanocarriers (AFDNs), based on ApDCs, encapsulate drugs for protection and exhibit specific chemical modifications. AFDNs are not only helpful to improve drug solubility, ability to cross various biological barriers, and circulation time in vivo, but also achieve highly specific targeting with the help of aptamers. Common nanocarrier systems, including micelles, liposomes, metal nanoparticles, and solid lipid nanoparticles, demonstrate well-established fabrication protocols, yet often face challenges with in vivo stability. Emerging nanoplatforms, such as four-way junction RNA nanostructures [106] and stimuli-responsive drug delivery systems [107], represent significant advances toward enhanced therapeutic efficacy and precision medicine. However, most nanomedicines still require intravenous injection [108], and there is no precedent for AFDNs in EC clinical trials; however, this advanced drug delivery system may be an important means to treat EC and other diseases in the future.

**Figure 5:**
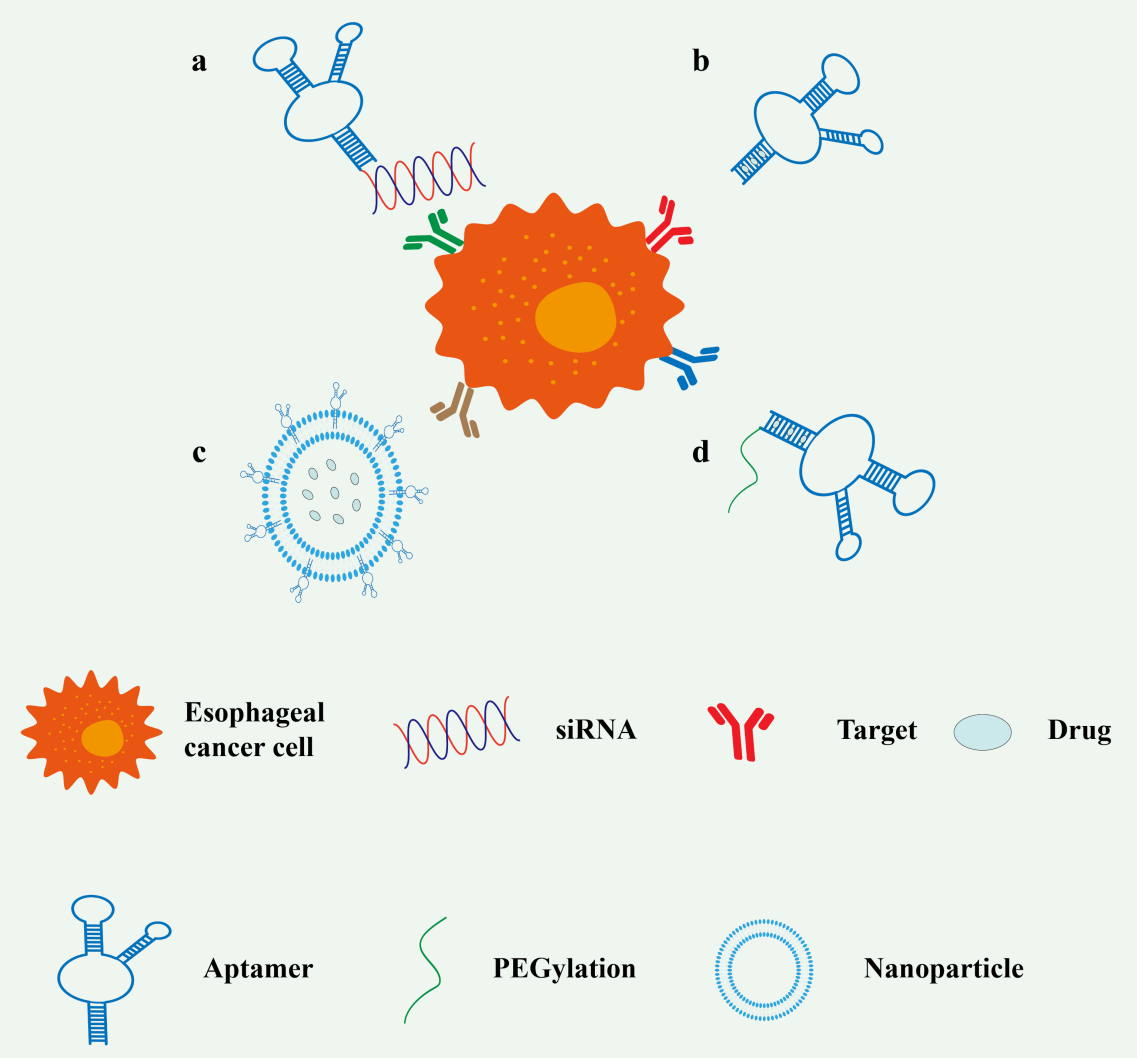
Aptamers serve as the primary means of carrier in drug delivery systems. (a) Aptamers coupled to siRNA. (b) Aptamers directly coupled to a chemotherapeutic drug (intercalation). (c) Aptamers and chemotherapeutic drug co-encapsulated in nanoparticles. (d) PEGylated aptamers improve drug stability.

To enhance delivery efficiency and therapeutic outcomes, researchers have developed various aptamer–drug conjugation strategies, primarily including: (1) covalent conjugation of aptamers with siRNA for precise delivery of gene-silencing therapeutics; (2) direct intercalation of aptamers with small-molecule chemotherapeutic drugs (e.g., DOX), forming stable complexes; (3) co-encapsulation of aptamers and hydrophobic drugs into nanoparticles (e.g., liposomes or polymeric micelles) to improve drug solubility and tumor targeting; and (4) PEGylation of aptamers, where polyethylene glycol (PEG) is covalently linked to aptamers, typically via amino, thiol, or carboxyl groups introduced at the 5′ or 3′ end (Figure 5). This PEGylation increases the molecular weight of the drug complex, potentially exceeding the renal threshold (30–50 kDa), thereby prolonging circulation half-life and enhancing stability. These strategies collectively improve drug bioavailability while reducing off-target toxicity, offering versatile solutions for aptamer applications in precision medicine. The following sections give a summary of the literature on drugs directly modified with aptamers and aptamer-functionalized drug nanocarriers for drug delivery in esophageal cancer (Table 3).

**Table 3:** Aptamer drug delivery systems against esophageal cancer.

Target	Aptamer	Sequence (5'-3')	Target cell line	Preclinical model	Refs.

unknown	Te4	AGC CTA AGC CTG TCC AGG AAT CGT GGT GGG GCA TCT CGC GAA ATT TGC TGA ACT GGT CAG TGG ATG GCT TAG TGG CAC GAT TAG GTC	TE-1	BALB/c nude mice	[113]
prohibitin 2	SYL-6	CTA GGA GTG GTT ATA AGG CGT AGG GGA AGG CGG GTC CAG G	KYSE450	BALB/c nude mice	[114]
integrin β1	A2	CAC CAC GCG AAT GCT ATC GGG GCT AAG TAT CAA AAT GAG C	KYSE410	BALB/c nude mice	[116]
integrin β1	A2 (35)	CGC GAA TGC TAT CGG GGC TAA GTA TCA AAA TGA GC	KYSE410	–	[117]
nucleolin	AS1411	AAA AAA GGT GGT GGT GGT TGT GGT GGT GGT GG	KYSE520 and KYSE70	BALB/c nude mice	[121]
EFNA1	EA1	AGC CTA AGC CTG TCC AGG AAT CGA TGG CTT AGT GGC ACG ATT AGG TCA GGA ATC GAT GGC TTA GTG GCA CGA TTA GGT C	KYSE150	BALB/c nude mice	[126]
EGFR	EGFR aptamer	Gcc uuA GuA AcG uGc uuu GAu ucGAcA GGA GGc lower cases indicate 2′-F nucleotides	KYSE150	BALB/c nude mice	[106]
ATP	ATP aptamer	ACC TGG GGG AGT ATT GCG GAG GAA GG	KYSE70 and EC109	–	[107]

#### 5.1 Aptamer–drug conjugates

The anthracycline drug doxorubicin serves as a first-line therapeutic agent for esophageal carcinoma, yet its clinical efficacy is frequently compromised by acquired drug resistance. Studies have revealed that one pivotal mechanism underlying this resistance involves the overexpression of P-gp on tumor cell surfaces [109]. Notably, DOX has been widely employed in drug delivery systems [110–111] due to its unique ability to spontaneously intercalate into GC-rich regions of aptamers through non-covalent interactions. This characteristic not only enhances tumor-specific accumulation but also significantly reduces cardiotoxicity. This way of inserting the drug directly between the bases of the aptamer and forming physical conjugation with the aptamer is called intercalation. It should be noted that there is a problem of drug leakage in advance in the actual application of intercalation. The solution strategies mainly include chemical modification of aptamers and modification of nanocarriers [112]. Jin et al. [113] screened the single-stranded DNA aptamer Te4, which specifically binds to TE-1 cells, through Cell-SELEX and further formed aptamer–DOX complexes to deliver DOX directly to cancer cells. In vivo imaging showed that the fluorescence signal of the complex weakened after 150 min, and the circulation time was short, which may be due to the fact that the aptamer was not modified and was easily degraded by nucleases. The cytotoxicity of Te4 to TE-1 and HEEC cells showed no potential as a therapeutic agent for EC alone. In addition, Te4 target has been identified as a membrane protein only by flow cytometry, and the specific target has not been extracted and identified. Qiao et al. [114] screened the SYL-6 aptamer with the strongest binding ability to ESCC cells by Cell-SELEX, identified and verified PHB2 as the molecular target for SYL-6-specific binding. The results of mouse model showed that SYL-6-Dox had almost the same antitumor activity as free Dox in vivo. In order to further enhance the antitumor effect, the use of nanotechnology to improve the stability of SYL-6 is key. It is worth noting that the SYL-6 aptamer also yields strong fluorescence signals on colon cancer and other tissues, indicating that SYL-6 can be used as a universal probe for detecting various cancers.

Integrin β1 is overexpressed in ESCC and interacts with L1CAM to activate the AKT signaling pathway, mediating ESCC drug resistance [115]. Zhang et al. [116] used Cell-SELEX to develop the DOX-loaded aptamer A2 (80), which can target integrin β1, where the ratio of A2-NA to DOX can reach at least 1:100. Although A2-NA-Dox has high affinity and specificity for ESCC tissues, its efficacy is slightly lower than that of free DOX. This may be due to a variety of reasons, including structural complexity and oversize resulting in low cellular uptake, poor stability due to tumor microenvironment, and inadequate drug release due to inadequate drug release mechanisms. Two years later, a novel bivalent aptamer-DNA-Dox conjugate (BADD) [117], was developed, and the A2 aptamer was truncated to A2 (35) to reduce steric hindrance and improve specific binding. A2 (35) also targets integrin β1 with an optimized Dox/BADD ratio of 5:1 to specifically kill tumor cells. The advantage of BADD is that the construction method is simple and rapid, but its biological stability may not be sufficient to meet the clinical needs of long circulation in vivo. Multivalent aptamers have more binding sites and thus higher affinity [118–119]. If further coupling negatively charged nanoparticles or PEG modification is carried out, nuclease hydrolysis can be avoided and stability can be improved. It should be noted that the PEG strategy should select the appropriate PEG molecular weight, localize PEG at noncritical sequences of the aptamer, and reduce the effect of steric hindrance on the aptamer binding ability.

AS1411 spontaneously folds to form a stable G-quadruplex structure, enabling it to efficiently and specifically recognize and bind to nucleolin overexpressed on cell surfaces; it can be directly used as a wide-range tumor therapeutic [120]. Zheng et al. [121] developed a novel aptamer conjugate drug that utilizes AS1411 aptamer targeting nucleolin to achieve targeted therapy against EC cells by binding AS1411 to the apolipoprotein portion of human serum albumin and lidamycin and loading it with the active enediyne chromophore (HSA-LDP-AE). The compound can effectively reduce nucleolin levels, specifically induce apoptosis of esophageal cancer cells, and significantly inhibit the growth of tumor tissues. This study provides a potential therapeutic strategy for esophageal cancer and demonstrates the potential application of aptamer technology in esophageal cancer treatment.

#### 5.2 Aptamer-functionalized drug nanocarriers

A multifunctional strategy based on aptamer nanoformulation of chemotherapeutic drugs and gene therapy could protect chemotherapeutic drugs from clearance, improve their accumulation at tumor sites, and potentially lead to more positive clinical feedback. Paclitaxel (PTX) was approved by the FDA in 1992 for the treatment of a variety of solid tumors. Poor water solubility is a major obstacle limiting its anticancer activity. PTX dominated the market for many years until the arrival of Abraxane, an albumin-based formulation of paclitaxel. Abraxane is the gold standard for cancer treatment, and the formulations developed by researchers can be directly compared head-to-head with Abraxane. Related reports include chimeric polypeptide–PTX nanoparticles [122], poly-(γ-ʟ-glutamine)–PTX nanoparticles [123], and docetaxel–carboxymethylcellulose nanoparticles [124]. Nevertheless, these preparations have been reported to have toxic side effects similar to PTX [125]. Xie et al. [126] screened EA1 aptamers by Cell-SELEX, which were used to deliver PTX and siRNA to ESCC cells overexpressing EFNA1. Due to the excellent permeability and retention of natural yolk lipid nanocarriers (EYLNs) [127], as well as the combined effects of chemotherapy and gene therapy, the EA1-EYLNs-PTX/siEFNA1 delivery system exhibited significant antitumor effects, which were verified in cell experiments, subcutaneous transplanted tumors and ESCC mouse models with lung metastasis. Li et al. constructed a four-way linked RNA nanocarrier to reduce the non-tumor tissue distribution effect of most nanocarriers during drug delivery, and to exploit the significant advantages of high thermal, pH, and enzyme stability of RNA nanocarriers [106]. Li et al. first confirmed abnormal expression of miR-375 and EGFR in ESCC tissues, and modified EGFR aptamers on the surface of RNA nanovectors to achieve high loading and active targeting of miR-375 and PTX. MiR-375 significantly inhibited ESCC cell proliferation, migration, and invasion by inducing apoptosis, regulating cell cycle and inhibiting epithelial–mesenchymal transition; also it exhibited no significant toxicity to heart, liver, or other major organs. The safety and effectiveness of this four-way connected nanostructure has also been verified by other researchers in the treatment of triple negative breast cancer [128].

Scientists are also interested in intelligent drug delivery systems that control drug release by using pH [129], enzymes [130], hypoxia [131], or ATP as triggers to achieve on-demand therapy. Wang et al. [107] designed a PEI–aptamer–EPI nanoplatform that relies on high ATP concentrations to release epirubicin (EPI). The polyethyleneimine (PEI) coating makes the whole delivery system positively charged, enables better adsorption to cell membranes, and promotes internalization. In vitro release experiments showed that about 50% of EPI was released after 25 h. Cytotoxicity studies were carried out in KYSE-70 and EC109 cell lines. PEI–aptamer–EPI had significantly higher tumoricidal effects compared with EPI and Aptamer-EPI groups. Unfortunately, this study was only in vitro; biocompatibility, in vivo targeting ability, and tumor inhibition efficiency of the nanosystem need to be further verified through in vivo experiments.

### 6 Discussion

Aptamers not only have a low incidence of Treatment Adverse Events (TAEs), but also can use antidote oligonucleotides to precisely regulate the aptamer functions. Therefore, aptamer applications are relatively safe. However, it is necessary to avoid using high-molecular-weight PEG modification as much as possible to avoid serious immune response.

At present, the clinical diagnosis method of EC is mainly endoscopic screening, which may cause discomfort to patients, and requires expensive equipment for detection. Aptamer-based hybrid sandwich biosensors reduce the probability of false negative diagnoses caused by only detecting a single biomarker, enable minimally invasive or non-invasive sample collection, and can use readily available clinical samples such as blood and urine for detection. However, in the early stages of EC, the concentration of biomarkers in body fluid samples may still be at low levels, and using multiple biomarker combinations to detect and construct biomarker panels is a better strategy to improve diagnostic accuracy.

Therapeutic aptamers, such as P42, P58, ZY3A and SPP1 aptamers, mostly exert pharmacological effects on the principle of target inhibition. ZY3A targets the membrane protein p37, and SPP1 belongs to secreted proteins. SOX2 and SOX2/CDP interfaces, the targets of P42 and P58, exist in cells, requiring aptamer penetration. Cell-penetrating peptides can improve aptamer transport by triggering adsorption-mediated endocytosis and may be an alternative strategy to solve this puzzle. For example, Le et al. [132] constructed nanocomplexes promising for the treatment of hepatocellular carcinoma by double modification of the cell-penetrating peptide iRGD and the aptamer EpCAM, and thus demonstrated optimal antitumor effects in vitro and in vivo experiments.

Three of the articles cited in this review utilized nanotechnology to form ApDCs. Among them, the parameters to be improved include optimizing the formulation, precisely controlling the size and distribution of nanoparticles by microfluidic devices, constructing composite nanocarriers, using 2′-O-Me, 2′-F, or locked nucleic acid modification as well as PEG modification to enhance the enzymatic resistance and stability of aptamers, and optimizing the sequence. In 2022, Zhu et al. [133] proposed the new concept of “drugtamers”, aiming to replace four clinically approved nucleoside analogs with four typical aptamers, achieving a drug loading rate of 100%. This approach not only retains the aptamers’ highly specific binding ability, but also confers additional pharmacological activity. Unfortunately, this article is missing the part about setting up aptamer groups as controls in mouse tumor models.

However, the clinical translation and regulatory approval of aptamers for cancer diagnosis, therapy, and drug delivery face multiple challenges. First, aptamers are susceptible to degradation by nucleases or proteases and rapid renal clearance, making oral administration particularly challenging. This necessitates regulatory agencies to develop tailored evaluation criteria, such as extending observation periods beyond those for conventional small-molecule drugs, incorporating specific assessments for immunotoxicity, organ toxicity, and nanoparticle accumulation in organs. Second, the negatively charged phosphate backbone of nucleic acid aptamers generates electrostatic repulsion with the anionic phospholipid bilayer of cell membranes, significantly limiting their cellular uptake. While cationic nanocarriers can effectively mediate delivery, this strategy substantially increases manufacturing complexity and production costs. Regulatory bodies must therefore establish new quality control standards to ensure batch-to-batch consistency. Third, approved aptamer-based therapies remain scarce in oncology, with no such treatments having received full regulatory approval to date. The most notable case is the AS1411 aptamer, which demonstrated only limited antitumor activity in a phase-II clinical trial for metastatic renal cell carcinoma [134]. The observed objective response rate failed to meet the predefined efficacy threshold, ultimately leading to regulatory rejection.

To improve aptamer permeability and enzyme resistance, and to enable clinical transformation of potentially useful aptamers, there are two strategies. First, chemical modification of aptamers. By truncating redundant sequences, not only the cost of research and development is reduced, but also the specificity of aptamers is enhanced. In addition, introducing locked nucleic acid modification, thiophosphate backbone substitution, and 3′ or 5′ capping of aptamers are also important strategies to optimize aptamer performance [135]. The second strategy is encapsulation of nanocarriers, such as metal nanoparticles, quantum dots, silica nanoparticles and liposomes. Gold nanoparticles (AuNPs) have different morphologies, among which gold nanospheres are the most popular. Spherical AuNPs are synthesized by using the Turkevich method [136], which is the most commonly used method. Although this method is time-consuming and somewhat cytotoxic, it is simple, low-cost, and easy to implement. AuNPs can be coupled to PEG for surface modifications. In conclusion, aptamers show great potential in the comprehensive management of EC and may provide more efficient and safer diagnosis and treatment for EC patients.

## Conclusion

The prognosis of patients with advanced esophageal cancer is poor, the treatment effect is poor, the efficacy of single-agent chemotherapy is limited, and clinical studies of multiple drugs have not shown meaningful results for advanced EC. Combination chemotherapy has been the reference first-line treatment for advanced EC for a long time, but the effect is not satisfactory. Targeted therapy and immunotherapy have less toxicity to normal cells and greatly improve the complete response rate of patients. Aptamers can be used not only as diagnostic probes for esophageal cancer, but also as carriers for therapeutic agents and drug delivery systems. By coupling with antitumor drugs and nanocarriers, aptamers can improve the targeted delivery efficiency of drugs and reduce non-specific distribution, thus enhancing the effectiveness of traditional chemotherapy. However, the complex processes involved in drug delivery systems and competition with targeted drugs make it difficult to translate into clinical decisions. With the accumulation of clinical data and technological developments, the development of safe and efficient aptamer-based biosensors, therapeutic agents, and drug delivery systems is imminent.

## Data Availability

Data sharing is not applicable as no new data was generated or analyzed in this study.
